# Implications for evolutionary trends from the pairing frequencies among golden‐winged and blue‐winged warblers and their hybrids

**DOI:** 10.1002/ece3.6717

**Published:** 2020-09-24

**Authors:** John L. Confer, Cody Porter, Kyle R. Aldinger, Ronald A. Canterbury, Jeffery L. Larkin, Darin J. Mcneil

**Affiliations:** ^1^ Department of Biology Ithaca College Ithaca NY USA; ^2^ Department of Zoology and Physiology University of Wyoming Laramie WY USA; ^3^ Wildlife Biology Program Lees‐McRae College Banner Elk NC USA; ^4^ West Virginia Cooperative Fish and Wildlife Research Unit, Division of Forestry and Natural Resources, West Virginia University Morgantown WV USA; ^5^ Department of Biological Sciences University of Cincinnati Cincinnati OH USA; ^6^ Department of Biology Indiana University of Pennsylvania Indiana PA USA; ^7^ Department of Entomology The Pennsylvania State University University Park PA USA

**Keywords:** backcrossing, behavioral isolationFCS express image cytometry, Blue‐winged Warblers, Golden‐winged Warblers, hybrid fitness, speciation

## Abstract

Extensive range loss for the Golden‐winged Warbler (*Vermivora chrysoptera*) has occurred in areas of intrusion by the Blue‐winged Warbler (*V. cyanoptera*) potentially related to their close genetic relationship. We compiled data on social pairing from nine studies for 2,679 resident *Vermivora* to assess evolutionary divergence. Hybridization between pure phenotypes occurred with 1.2% of resident males for sympatric populations. Pairing success rates for Golden‐winged Warblers was 83% and for Blue‐winged Warblers was 77%. Pairing success for the hybrid Brewster's Warbler was significantly lower from both species at 54%, showing sexual selection against hybrids. Backcross frequencies for Golden‐winged Warblers at 4.9% were significantly higher than for Blue‐winged Warblers at 1.7%. More frequent backcrossing by Golden‐winged Warblers, which produces hybrid phenotypes, may contribute to the replacement of Golden‐winged by Blue‐winged Warblers. Reproductive isolation due to behavioral isolation plus sexual selection against hybrids was 0.960. Our analyses suggest that plumage differences are the main driving force for this strong isolation with reduced hybrid fitness contributing to a lesser degree. The major impact of plumage differences to reproductive isolation is compatible with genomic analyses (*Current Biology*, 2016, 26, 2313), which showed the largest genetic difference between these phenotypes occurred with plumage genes. These phenotypes have maintained morphological, behavioral, and ecological differences during two centuries of hybridization. Our estimate of reproductive isolation supports recognition of these phenotypes as two species. The decline and extirpation of the Golden‐winged Warbler in almost all areas of recent sympatry suggest that continued coexistence of both species will require eco‐geographic isolation.

## Graphical abstract

Of 2,679 Vermivora in breeding habitat, we document that only 1.2% of the social pairs involved a golden wing × blue wing cross, and hybrids had significantly reduced pairing success. Together, these factors showed a reproductive isolation of 0.960, sufficient to recognize Golden‐winged and Blue‐winged Warblers as two species. This strong assortative mating is related to plumage differences, which in turn is related to genomic analyses that showed strong differences in regions related to plumage and coloration.

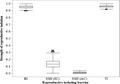

## 1 INTRODUCTION

A central goal of evolutionary biology is to elucidate the processes that generate and maintain the diversity of life, especially those responsible for the origin of species. Evolutionary biologists have long recognized that understanding how reproductive isolating barriers evolve and reduce gene flow between diverging lineages is essential for understanding the origin of species (Sobel, Chen, Watt, & Schemske, 2010). While a number of methodological approaches are used to study speciation, most provide only indirect information on the isolating barriers and evolutionary mechanisms driving speciation (Coyne & Orr, 2004). Consequently, there has been a growing call for more studies that directly estimate the degree to which different isolating barriers reduce gene flow between diverging lineages in nature (Coyne & Orr, 2004; Schemske, 2010; Sobel et al., 2010).

One especially powerful approach for understanding the mechanistic basis of speciation is to estimate the strength of reproductive isolating barriers between sympatric lineages that are incompletely reproductively isolated (Coyne & Orr, 2004; Nosil, 2012; Sobel et al., 2010; Sobel & Streisfeld, 2015). By focusing on lineages that have not yet evolved complete reproductive isolation, one can identify the isolating barriers that reduce gene flow and thus contribute to speciation, as opposed to barriers that evolve after speciation (Coyne & Orr, 2004; Sobel & Streisfeld, 2015). Such an approach can be particularly informative if the lineages in question have been well‐characterized genomically. Because historical and ongoing gene flow may homogenize neutral regions of the genome, the genomic regions and traits that contribute to reproductive isolation can be distinguished (Poelstra et al., 2014). Subsequently, the strength of isolating barriers hypothesized to be affected by these traits can be estimated, allowing evaluation of causal links between genomic divergence, trait divergence, reproductive isolating barriers, and ultimately speciation (Seehausen et al., 2014).

Here, we investigate the strength of reproductive isolating barriers in a pair of closely related bird species. Previous work on Golden‐winged and Blue‐winged Warblers found 3% sequence divergence in the mitochondrial (mtDNA) genomes of these species, with the contemporary distribution of mtDNA lineages corresponding to allopatric populations (Gill, 1997, 2004; Shapiro, Canterbury, Stover, & Fleischer, 2004). These data suggest that divergence between Golden‐winged and Blue‐winged warblers was initiated in allopatry roughly 1.5 million years ago (Gill, 2004; Weir & Schluter, 2008). In contrast to these high levels of mtDNA divergence, recent whole‐genome sequencing revealed very low divergence in the nuclear genome, with only six small regions showing strong divergence (Toews et al., 2016). Therefore, despite having diverged in allopatry roughly 1.5 million years ago (close to the ~2 million years of geographic isolation required, on average, for bird speciation; Price, 2008) and current high levels of geographic isolation and morphological divergence, introgression between Golden‐winged and Blue‐winged Warblers in the zone of recent sympatry appears to be high.

Toews et al. (2016) noted that of the six genomic regions that are highly divergent between Golden‐winged and Blue‐winged Warblers, and four were identified as being involved in feather development or pigmentation. Consequently, reproductive isolating barriers affected by plumage divergence (i.e., those related to mate choice) may be quite strong in this system. Indeed, strong reproductive isolation based on plumage differentiation may be a primary mechanism that has maintained the distinctiveness of these lineages, especially since there appear to be no intrinsic or ecologically based reductions in hybrid fitness (Vallender, Friesen, & Robertson, 2007).

Most field studies of interbreeding by Golden‐winged and Blue‐winged Warblers have omitted quantitative analyses of reproductive isolating barriers, in part due to low sample sizes of social, hybrid pairs. Some studies have found evidence for behavioral isolation (Confer & Larkin, 1998; Ficken & Ficken, 1968b) and sexual selection against hybrids (Confer & Tupper, 2000; Ficken & Ficken, 1968a, 1968b; Leichty & Grier, 2006), while others have not (Vallender et al., 2007). We address this uncertainty by compiling data from nine published studies across eight localities on social pairs of Golden‐winged Warblers, Blue‐winged Warblers, and their hybrids. This comprehensive dataset allowed us to provide robust estimates of the strength of behavioral isolation and sexual selection against hybrids: the two reproductive isolating barriers that should be directly tied to the plumage and genomic divergence between these species. We further test the effect of plumage divergence on reproductive isolation by quantifying the relationship between plumage divergence and the frequency with which individuals of the two parental species and their hybrids form social pairs.

## 2 METHODS

We compiled data on social pairs from studies published by five of the authors. In addition, we included data from Ficken and Ficken (1968a, 1968b), and from Will (1986) with supplemental data from Will (*personal communication*). This provided a total of nine, chronologically distinct studies in eight study areas. For each study area and for pooled data, we compiled the frequency of social pairing for Golden‐winged and Blue‐winged Warblers and hybrid phenotypes. Not all studies could be used for all calculations because of limitations in the recorded data.

### 2.1 Study areas

#### 2.1.1 Old field succession in Tompkins County, New York

Ficken and Ficken (1968a, 1968b) compiled phenotypic pairing frequencies and pairing success rates for *Vermivora* spp. during four seasons spanning 7 years (1961–1966). The habitat was a single successional site with an elevation range of 284–315 m.

#### 2.1.2 Old field succession in Midland County, Michigan

Will (1986) monitored pairing by *Vermivora* spp. for 3 years (1982–84) within old field habitat. The study area consisted of one site with an elevation range of 205–209 m. We compiled pairing success frequencies for his study using data from Will (1986) and supplemental information (Will, *personal communication*).

#### 2.1.3 Old field succession in Oswego County, New York

Confer and Larkin (1998) described pairing frequencies by *Vermivora* spp. over seven consecutive years (1988–1994) across 21 sites where elevation ranged from 80 to 130 m. The sites provided dry successional habitat although some predominately dry sites included adjacent ephemeral wetlands. Unpaired birds were not determined for this study and these results could not be used to calculate pairing success rates.

#### 2.1.4 Diverse habitats in Orange County, New York (1998–1999)

Confer and Tupper (2000) observed pair formation for resident, male Golden‐winged and Brewster's Warblers in Sterling Forest State Park. Study sites (*n* = 6) ranged in elevation from 200 to 350 m and included utility rights‐of‐way and other successional habitats. Data from this study were insufficient to calculate pairing success rates or hybridization for male Blue‐winged Warblers, but were used to calculate the frequency of primary hybridization and the frequency of backcrossing by Golden‐winged Warblers.

#### 2.1.5 Diverse habitats in Orange County, New York (2001, 2003–2006, 2008)

Confer, Barnes, and Alvey (2010) studied *Vermivora* spp. pairing in a variety of habitats in southern New York within Sterling Forest State Park. The habitats monitored included swamp forests, shrub swamps, managed utility rights‐of way, and successional habitat. In total, 25 sites were monitored ranging in elevation from 200 to 350 m.

#### 2.1.6 Lightly grazed pastures in Randolph and Pocahontas Counties, West Virginia

Phenotypic pairing frequencies and pairing success rates were monitored at 14 sites during 2008–2014 in grazed pastures described by Aldinger et al., 2014, Aldinger, 2018. Sites were at 800–1,000 m elevation in Randolph County and at 700–1,250 m in Pocahontas County.

#### 2.1.7 Managed forest in Pike and Monroe Counties, Pennsylvania

In Pennsylvania's Delaware State Forest, *Vermivora* spp. pairing was monitored across seven managed forest sites ranging from 400 to 550 m in elevation from 2012 to 2014. Habitats were created via overstory removal timber harvest and described in detail by McNeil et al., 2017, 2018).

#### 2.1.8 Abandoned farmland and pastures in Mercer County, West Virginia

Canterbury (2012) compiled phenotype pairings by *Vermivora* spp. in abandoned farmland and lightly grazed pastures from 2001 to 2009 at four sites. These sites occurred at 700–900 m in elevation.

#### 2.1.9 Abandoned coal mines in Wyoming and Raleigh Counties, West Virginia

Phenotype pairings by breeding *Vermivora* spp. were compiled for six strip‐mined sites at 700–1,000 m elevation from 2003 to 2012 as described by Canterbury (1990), Canterbury, Stover, and Nelson (1993), Canterbury and Stover (1999) and Shapiro et al. (2004).

### 2.2 Hybrid phenotypes and genotypes

**FIGURE 1:**
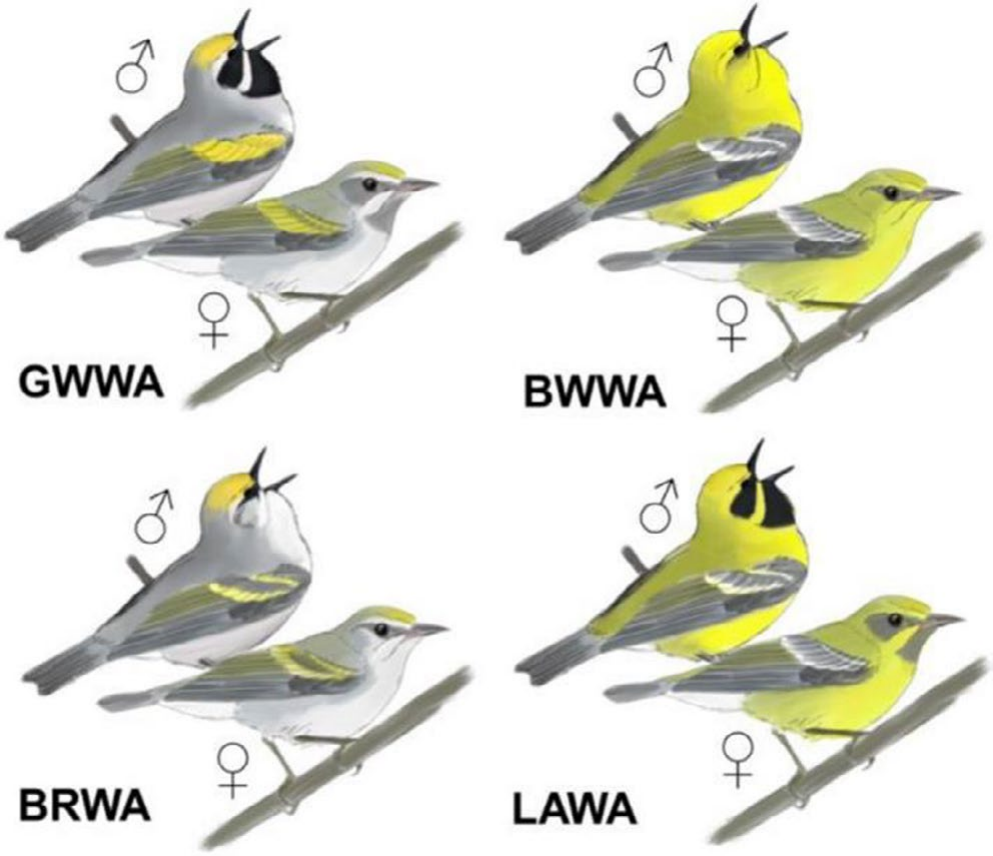
*Vermivora* spp. phenotypes considered in this study: Golden‐winged Warbler (*V. chrysoptera*; GWWA), Blue‐winged Warbler (*V. cyanoptera*; BWWA), “Brewster's” Warbler (hybrid; BRWA) and “Lawrence's” Warbler (hybrid LAWA). Males (♂) and females (♀) are both shown. Original drawings by DJM

Following Parkes (1951), we consider two hybrid phenotypes, the Brewster's Warbler (*Vermivora leucobronchialis*, Brewster (1874)) and Lawrence's Warbler (*Vermivora lawrencii*, Herrick (1874)). Parkes described the color patterns as if they were due to two genes each having a dominant and a recessive allele. This two gene model provides a fairly accurate predictor of the pattern of phenotype inheritance (Toews et al., 2016), although it is insufficient to explain occasional intermediate phenotypes. According to Parkes’ model, Brewster's Warblers are the F1 product of primary hybridization between genetically pure Golden‐winged and Blue‐winged Warblers, but can also result from matings of other genotypes within the Golden‐winged and Blue‐winged Warbler complex. This phenotype is characterized by the contour plumage of a Golden‐winged Warbler with a gray back and white underside coupled with the facial pattern of a Blue‐winged Warbler (Figure 1). The Lawrence's Warbler has the body color of a Blue‐winged Warbler and the facial pattern of a Golden‐winged Warbler (Figure 1). In Parkes' model, the Lawrence's phenotype is homozygous recessive for both genes and can be produced by an F1 × F1 cross.

While there is a strong correlation between phenotype and genotype of individuals in this system (Toews et al., 2016), it is important to note that some phenotypically “pure” individuals show signs of introgression in their genetic background (Dabrowski, Fraser, Confer, & Lovette, 2005; Vallender et al., 2009; Wood et al., 2016). The presence of these “cryptic hybrids” will inflate our estimates of reproductive isolation (see below) and overestimate the reduction in gene flow due to a given barrier. Nonetheless, assortative mating by plumage phenotype and/or sexual selection against males with intermediate phenotypes would still act to reduce gene flow between lineages, thus promoting speciation. The main goal of this study was to determine whether there is nonrandom mating based on these phenotypic differences, and thus whether patterns of mating in the field are consistent with patterns of genomic divergence primarily in regions related to plumage development (Toews et al., 2016). We note that an imperfect relationship between phenotype and genotype is precisely what is expected in systems that are in the early stages of speciation (Dobzhansky, 1958; Roux et al., 2016) and thus not unique to Golden‐winged and Blue‐winged Warblers.

### 2.3 Residency, pairing, primary hybridization, and backcrosses

Males were considered as a resident at each study area if they were heard or seen on at least 3 days over a week's span of time within an area approximately the size of *Vermivora* spp. territories (e.g., Confer, Allen, & Larkin, 2003). Almost all males were seen over a much longer period. Following the methods of Will (1986) and others (Canterbury, 2012; Confer et al., 2003; Vallender et al., 2007), we considered males to have formed a pair with a female if they were observed feeding nestlings or fledglings or if they were seen on a perch close to the nest on several occasions. We considered pairing attributes for a banded male that returned to breed in another year as an additional, independent event.

Conspicuous singing with type 1 calls from one or a few song posts (Gill & Murray, 1972a) by paired or unpaired males provides a strong clue about the location of an established or desired breeding territory. After searching on three mornings for a total of at least six hours and spanning at least a week, a male was thought to be unpaired if no evidence of nesting was found near such song posts. Females are very cryptic, and almost all observed females were engaging in reproductive activities (e.g., nest building, carrying food, and alarm behavior). This provides a very biased sample of the proportion of females that are paired. Consequently, we estimated pairing success rates only for males. We quantified the pairing success rate at each study area as the fraction of the resident males that formed a social pair averaged for all years of each study. We equate primary hybridization to the formation of a social pair between phenotypes of Golden‐winged and Blue‐winged Warblers.

### 2.4 Estimating the strength of reproductive isolating barriers

We estimated the strength of one prezygotic reproductive isolating barrier (BI or behavioral isolation) and one postzygotic reproductive isolating barrier (SH or sexual selection against hybrids) based on the social pairing data. To estimate the strength of each barrier, we used the RI index of Sobel and Chen (2014). Specifically, behavioral isolation was estimated as.

(1) $$\text{BI}=1‐2\left[\frac{\frac{\text{HetObs}}{\text{HetExp}}}{\frac{\text{ConObs}}{\text{ConExp}}+\frac{\text{HetObs}}{\text{HetExp}}}\right]$$

where HetObs denotes the number of observed heterospecific social pairs, HetExp denotes the number of expected heterospecific social pairs assuming random mating, ConObs denotes the number of observed conspecific social pairs and ConExp denotes the number of expected conspecific social pairs assuming random mating. Because Golden‐winged and Blue‐winged Warblers differed in their relative abundance at our study sites, the random expectations for heterospecific pairing and conspecific pairing differ among sites. To correct for this, we incorporated the IPSI equation developed by Rolán‐Alvarez and Caballero (2000) into Equation 1. This equation uses data on all four possible pairwise social pairing combinations to calculate expected values of conspecific and heterospecific pairing. Behavioral isolation was only estimated relative to phenotypically pure Golden‐winged and Blue‐winged Warblers to evaluate the effectiveness of the phenotypic differences between these lineages as a prezygotic reproductive isolating barrier.

Sexual selection against hybrids, which we refer to as hybrid fitness, was estimated as.

(2) $$\text{SH}=1‐2\left[\frac{\text{Hyb}}{\text{Pur}+\text{Hyb}}\right]$$

where Hyb denotes the proportion of phenotypically hybrid males that formed a social pair with a female and Pur denotes the proportion of phenotypically pure males that formed a social pair.

These equations produce symmetrical values that represent the proportional reduction in gene flow relative to expectations under random mating (Sobel & Chen, 2014). A slope of two ensures that values of RI range from −1 to 1, with 1 denoting complete reproductive isolation. The strength of both reproductive isolating barriers was estimated for each population, and 95% confidence intervals for individual RI indices were estimated using bootstrap resampling with 1,000 replicates using the *boot* package (Canty & Ripley, 2015) in R version 3.3.3.

We used the methods outlined in Coyne and Orr (1989) and Ramsey, Bradshaw, and Schemske (2003) to estimate the absolute contribution of each sequentially and independently acting reproductive isolating barrier (AC) to total reproductive isolation resulting from BI and SH. Because behavioral isolation acts first, AC_BI_ = BI. The absolute contribution of sexual selection against hybrids (AC_SH_) equals SH(1‐AC_BI_). Total reproductive isolation is the sum of the absolute contributions of behavioral isolation and sexual selection against hybrids (AC_BI_ + AC_SH_).

## 3 RESULTS

We obtained data on breeding Golden‐winged and Blue‐winged Warblers and their hybrids from nine studies at eight study areas over 47 years of field work. The total sample provided information on 2,679 resident males and females for Golden‐winged (*n* = 1,852) and Blue‐winged Warblers (*n* = 667) and their hybrids (*n* = 160; Table 1).

**TABLE 1:** Phenotype frequencies for resident Golden‐winged and Blue‐winged Warblers and their hybrids at each study area.

Sites	Golden‐winged Warbler		Blue‐winged Warbler		Percentage GWWA^a^	Brewster's Warbler		Lawrence's Warbler		Sum
	Male	Female	Male	Female		Male	Female	Male	Female	
Managed forest, high elevation PA	137	100	4	2	97%	12	6	0	0	261
Pasture, high elevation WV	245	180	14	7	95%	19	6	0	3	476
Old field, low to mid‐elevation WV	108	109	14	16	88%	5	2	0	0	254
Old field, north‐central NY	86	76	30	32	72%	4	12	1	1	242
Diverse habitats, s NY, 1998–1999	62	37	‐	‐	‐	13	1	0	0	113
Diverse habitats, s NY, 2009–2010	52	43	26	18	68%	3	2	0	0	144
Mine lands, mid‐elevation WV	298	267	246	185	57%	27	21	2	7	1,053
Old field, north‐central MI	26	13	23	15	51%	3	0	0	0	80
Old field, central NY	7	6	15	20	27%	8	2	0	0	58
Pooled observations	1,021	831	372	295	74%	94	52	3	11	2,679

^a^ Resident male and female Golden‐winged Warbler phenotypes as a percentage of the sum of resident Golden‐winged and Blue‐winged Warbler phenotypes.

### 3.1 Behavioral isolation

**FIGURE 2:**
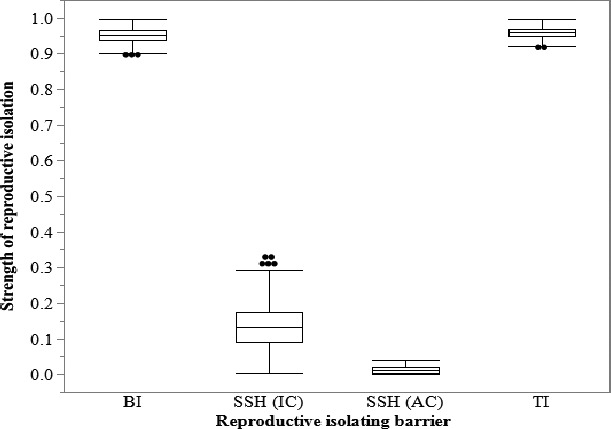
Box and whisker plot of the strength of reproductive isolating barriers (1,000 bootstrapped averages). Abbreviations denote the following: behavioral isolation (BI), individual component of sexual selection against hybrids (SSH (IC)), absolute contribution of sexual selection against hybrids (SSH (AC)), and total reproductive isolation (TI)

Across all studies, primary hybridization occurred with 0.9% (*n* = 14) of the 1,680 paired Golden‐winged Warblers. The tabulation and calculations of paired individuals (Table 2) include 13 bigamous males that paired with 24 female Golden‐winged Warblers and two Brewster's Warblers. Also included is one female Golden‐winged Warbler predated on her nest before her mate was identified. Primary hybridization occurred with 2.4% (*n* = 14) of the 583 paired Blue‐winged Warblers, substantially less primary hybridization than expected by chance for either species (Table 2; chi‐square with Yates correction: *χ* ^2^ = 7.47, *p* = 0.006). Collectively, 1.2% of the birds with a pure phenotype in these sympatric populations paired with the alternative pure phenotype (Table 2). These patterns reflect high levels of behavioral isolation, which ranged from 0.860 to 1 across our study sites (mean: 0.951; lower 95% CI: 0.902; upper 95% CI: 0.982). See Figure 2. We found no evidence for a relationship between the strength of behavioral isolation and the difference in relative abundance of Golden‐winged and Blue‐winged Warblers across sites (*r* ^2^ = 0.03, *df* = 7, *p* = 0.67), suggesting that the strength of behavioral isolation (the observed rate of interbreeding, corrected for random expectations) is unaffected by variation in local relative abundance of the two lineages.

**TABLE 2:** Numbers of paired Golden‐winged (GWWA) and Blue‐winged Warblers (BWWA) individuals for each study area, the proportion of paired individuals that were GWWA, and the number and frequency of primary hybrid pairs.

Study area	Paired GWWA^a^	Proportion of paired GWWA^c^	Paired BWWA^b^	Primary hybridization (n)^d^	Primary hybridization (%)^d^
Managed forest, high elevation, PA	198	5	98%	3	3.0%
Pasture, high elevation WV	357	15	96%	3	1.6%
Old field, low to mid‐elevation WV	217	30	88%	1	0.8%
Old field, north‐central NY	162	62	72%	3	2.7%
Diverse habitats, s NY, 1998–1999	74	—	—	4^e^	4.0%
Diverse habitats, s NY, 2009–2010	86	37	70%		
Mine lands, mid‐elevation WV	547	370	60%	0	0.0%
Old field, north‐central MI	26	29	47%	0	0.0%
Old field, central NY	13	35	27%	0	0.0%
Total	1,680	583	73%	14	
Pooled mean					1.2%
Mean of study areas					1.5%

^a^ Number of paired, male and female Golden‐winged Warblers.

^b^ Number of paired, male and female Blue‐winged Warblers.

^c^ Paired GWWA / (Paired GWWA + Paired BWWA)

^d^ Proportion and number of primary hybrid pairs out of all pairs Golden‐winged and Blue‐winged Warblers.

^e^ Value derived from the sum of primary hybrid pairings of Golden‐winged Warblers in 1998–'99 and primary hybrid pairings for Golden‐winged and Blue‐winged warblers in 2009–'10.

### 3.2 Sexual selection against hybrid males

For pooled values, the pairing success rate for male Golden‐winged Warblers was 83% and for male Brewster's Warblers was 54% (Table 3), a highly significant difference (*χ* ^2^ = 44.35, *p* < .0001). The pairing success rate for pooled values for male Blue‐winged Warblers at 77% also was highly significantly greater than for male Brewster's Warblers (Table 3; *χ* ^2^ = 19.16, *p* < .0001). The strength of sexual selection against hybrids varied across study areas (range: 0–0.409). Some of the variance would be due to random variation especially with the very small sample of hybrids in each study. To account for potential real differences in pairing success from one study area to another, we used paired *t* tests. With the differences paired by study area, male Golden‐winged Warblers had higher pairing success than male Brewster's Warblers (Table 3; two‐tailed, paired *t* test: *df* = 8, *t* = 3.25, *p* = .012) as did male Blue‐winged Warblers (Table 3; two‐tailed, paired *t* test: *df* = 7, *t* = 2.53, *p* = .039). The proportion of male Brewster's Warblers that did not form a social pair (46%) is 2.7‐fold greater than for Golden‐winged Warblers (17%) and 2‐fold greater than for Blue‐winged Warblers (23%). Consistent with these data, we found evidence for sexual selection against hybrids overall (mean: 0.138; lower 95% CI: 0.045; upper 95% CI: 0.294). See Figure 2.

**TABLE 3:** Pairing success rates for male Golden‐winged, Blue‐winged, and Brewster's Warblers for nine study areas

Study areas	Male GWWA (*n*)	Male GWWA Pairing Success (%)	Male BWWA (*n*)	Male BWWA Pairing Success (*%*)	Male BRWA (*n*)	Male BRWA Pairing Success (%)
aged Forest, high elevation, PA	137	72%	4	75%	12	58%
Pasture, high elevation, WV	245	72%	14	57%	19	68%
Old field, low to mid‐elevation, WV	108	100%	14	100%	5	100%
Old field, north‐central NY	86	100%	30	100%	4	100%
Diverse habitats, s NY, 1998–1999	62	60%	‐	‐	13	8%
Diverse habitats, s NY, 2009–2010	52	83%	26	73%	3	33%
Mine lands, mid‐elevation, WV	298	94%	246	75%	27	48%
Old field, north‐central, MI	26	50%	23	61%	3	33%
Old field. central NY	7	100%	15	100%	8	75%
Total	1,021		372		94	
Pooled values		83%		77%		54%
Study area means		81%		80%		58%

### 3.3 Total reproductive isolation

The combined action of behavioral isolation plus sexual selection against hybrids results in strong reproductive isolation between Golden‐winged and Blue‐winged Warblers at all study sites, ranging from 0.882 to 1 (mean: 0.960; lower 95% CI: 0.928; upper 95% CI: 0.983). The individual contribution of behavioral isolation to total reproductive isolation was much greater than that of sexual selection against hybrids (two‐tailed, paired *t* test: *df* = 5, *t* = 9.89, *p* < .001). See Figure 2.

### 3.4 Relationship between plumage divergence and pairing frequency

Of 849 paired, male Golden‐winged Warblers, 4.9% (*n* = 42) paired with a female Brewster's Warbler (Table 4). This backcross frequency is 5.4 times greater than the rate of primary hybridization (*χ* ^2^ = 24.29, *p* < .0001). For the 288 paired, male Blue‐winged Warblers, 1.7% (*n* = 5) paired with a Brewster's Warbler. Backcross frequency by male Blue‐winged Warblers was less than but not significantly different from their frequency of primary hybridization (*χ* ^2^ = 3.48, *p* = .062). The frequency of backcrossing by male Golden‐winged Warblers with Brewster's Warblers was 2.9 times greater than the rate for male Blue‐winged Warblers (*χ* ^2^ = 4.81, *p* = .028). Sample sizes for Lawrence's Warblers were too small for statistical analyses.

**TABLE 4:** Backcross frequencies: social pairs by male Golden‐winged (GWWA) and Blue‐winged Warblers (BWWA) with femaleBrewster's Warbler (BR) and Lawrence's Warbler (LA) hybrids

Study area	Male GWWA	GWWA × BR		GWWA × LA		GWWA × BR + LA		Male BWWA	BWWA × BR		BWWA × LA		BWWA × BR + LA	
	*n*	%	*n*	%	*n*	%	*n*	*n*	%	*n*	%	*n*	%	*n*
Managed forest, high elevation PA	98	5.1%	5	0	0	5.1%	5	3	0	0	0	0	0	0
Pasture, high elevation WV	177	4.0%	7	0.6%	1	4.5%	8	8	12.5%	1	25.0%	2	37.5%	3
Old field, low to mid‐elevation WV	108	1.9%	2	0	0	1.9%	2	14	0	0	0	0	0	0
Old field, north‐central NY	86	12.8%	11	1.2%	1	14.0%	12	30	0	0	0	0	0	0
Diverse habitats, sNY, 1998–1999	37	0	0	0	‐	‐	‐	‐	‐	‐	‐	‐	‐	‐
Diverse habitats, sNY, 2009–2010	43	2.3%	1	0	0	2.3%	1	19	5.3%	1	0	0	5.3%	1
Mine lands, mid‐elevation WV	280	5.0%	14	2.5%	7	7.5%	1	185	1.6%	3	0	0	1.6%	3
Old field, north‐central MI	13	0	0	0	0	0	0	14	0	0	0	0	0	0
Old field, central NY	7	8.6%	2	0	0	28.6%	2	15	0	0	0	0	0	0
Total	849		42		9		51	288		5		2		7
Pooled mean		4.9%		1.1%		6.1%			1.7%		0.7%		2.4%	
Study areas mean		6.6%		0.5%		7.1%			2.4%		3.1%		5.5%	

Of 834 paired, female Golden‐winged Warblers, 4% (*n* = 33) formed a backcross with a male Brewster's Warbler (Table 5), which is three times the rate of primary hybridization (*χ* ^2^ = 16.90, *p* = .012). For the 309 paired, female Blue‐winged Warblers, 3.6% (*n* = 11) formed a social pair with a Brewster's Warbler. Backcross frequency by female Blue‐winged Warblers was not significantly different than their frequency of primary hybridization (*χ* ^2^ = 0.888, *p* > .10). The frequency of backcrossing by female Golden‐winged Warblers with Brewster's Warblers was not different than the rate for female Blue‐winged Warblers (*χ* ^2^ = 0.0001, *p* > .10). Sample sizes for Lawrence's Warblers were too small for statistical analyses.

**TABLE 5:** Backcross frequencies: social pairs by female Golden‐winged (GWWA) and Blue‐winged Warblers (BWWA) with male Brewster's Warbler (BR) and Lawrence's Warbler (LA) hybrids

Study area	Female GWWA	GWWA × BR		GWWA × LA		GWWA × BR + LA		Female BWWA	BWWA × BR		BWWA × LA		BWWA × BR + LA	
	*n*	%	*n*	%	*n*	%	*n*	*n*	%	*n*	%	*n*	%	*n*
Managed forest, high elevation PA	100	6.0%	6	0	0	6.0%	6	2	0	0	0	0	0	0
Pasture, high elevation WV	180	6.7%	12	0	0	6.7%	12	7	14.3%	1	13.3%	2	26.7%	4
Old field, low to mid‐elevation WV	109	3.7%	4	0	0	3.7%	4	16	6.3%	1	0	0	3.3%	1
Old field, north‐central NY	76	1.3%	1	0	0	1.3%	1	32	6.3%	2	1.6%	1	4.5%	3
Diverse habitats, sNY, 1998–1999	37	0	0	0	0	0	‐	‐	‐	‐	‐	‐	‐	‐
Diverse habitats, sNY, 2009–2010	43	2.3%	1	0	0	2.3%	1	18	0	0	0	0	2.7%	3
Mine lands, mid‐elevation WV	267	3.0%	8	0	0	3.0%	8	185	0.5%	1	1.1%	4	1.6%	1
Old field, north‐central MI	13	0	0	0	0	0	0	15	6.7%	1	0	0	3.4%	1
Old field, central NY	6	16.7%	1	0	0	16.7% 1	1	20	25.0%	5	0	0	14.3%	5
Total	831		33		0		33	295		11		3		14
Pooled mean		4.0%		0	0	4.0%			3.7%		1.0%		4.7%	
Study areas mean		4.4%		0	0	4.4%			7.4%		0.45%		7.9%	

## 4 DISCUSSION

The degree of difference between Golden‐winged and Blue‐winged Warblers is difficult to quantify. In regions of sympatry, the two species often nest in old field successional habitat sometimes with overlapping territories. Yet, when specific habitats are available, the two phenotypes show strong differences in habitat preference. The managed forest in our study attracted 98% Golden‐winged Warblers (McNeil et al., 2017, 2018) despite being in a region dominated by Blue‐winged Warblers. Swamp forests in southern New York attracted 95% Golden‐winged Warblers (Confer et al., 2010) even though both species were about equally abundant in adjacent uplands. Rush and Post (2008) documented similar differences in habitat preference in a wetland‐upland mosaic in the St. Lawrence River valley. These examples support an intrinsic difference in breeding habitat preference. In addition, the winter range differs with Golden‐winged Warblers extending farther south into northern South America (Bennett et al., 2017; Kramer et al., 2017). Further, Blue‐winged Warblers arrive earlier on their sympatric breeding grounds (Canterbury & Stover, 1999; Ficken & Ficken, 1968a, 1968b). Golden‐winged Warblers weigh more and have larger wing chords, but smaller tarsi (Confer, 1992; Gill, Canterbury, & Confer, 2001). The primary song of Golden‐winged and Blue‐winged Warblers, which is used to attract mates, is readily distinguished (Ficken & Ficken, 1966, 1968a, 1968b,^ 1969^; Gill & Murray, 1972a; Highsmith, 1989) with small variation among males of the same phenotype (Gill & Murray, 1972b). Occasionally males alternate singing the primary song of one and then the other species (See references in Confer, Hartman, & Roth, 2020). During 47 years of field work and observation of nearly 1,137 paired males, the co‐authors observed 20 Golden‐winged Warblers and one Blue‐winged Warbler that demonstrated this bivalent singing. Notably, each song type seemed quite normal. Kramer et al. (2019) provide audio/visual documentation and analyses of this bivalent singing. These differences in habitat preference, range, behavior, song, and morphology surely have a genetic foundation, but their contribution of the differentiation between these two species is not readily quantified.

Our study provides a measure of the degree of speciation by compiling the pairing frequencies for sympatric populations of Golden‐winged and Blue‐winged Warblers and their hybrids. Among our nine studies at eight study areas some Golden‐winged Warbler populations were expanding, others semi‐stable, and others declining. Some study areas had a strong preponderance of Golden‐winged Warblers over Blue‐winged Warblers, others were semi‐equal, and others had a preponderance of Blue‐winged Warblers. Thus, our results represent a wide range of population conditions. Pooled values show a reproductive isolation of 0.960. Among all of these diverse populations (Table 2), the frequency of hybrid social pairs between the Golden‐winged and Blue‐winged phenotypes ranged from 0% for three studies to 4% (*n* = 4) in one study. Given the small sample size for hybrid pairs (*n* = 0–4), the variance of the frequency of hybridization among our study areas is compatible with the hypothesis that primary hybridization occurs with similar frequency among all sympatric populations.

An important limitation of using social pairing data to estimate the strength of behavioral isolation in birds is the presence of extra‐pair paternity, wherein either member of a pair may mate and produce offspring with an individual other than their social partner. Vallender et al. (2007) estimated that 32% of young in a contact zone between Golden‐winged and Blue‐winged Warblers were the result of extra‐pair paternity among the Golden‐winged Warblers. However, social pairing data should only produce biased estimates of behavioral isolation if individuals systematically seek extra‐pair partners that differ in phenotype from their social partner. Importantly, there was no evidence of extra‐pair paternity between Golden‐winged and Blue‐winged Warblers documented by Vallender et al. (2007). This suggests that behavioral isolation from social pairing data would be minimally confounded by the presence of extra‐pair or extra‐species copulations in this system.

Hybrid fitness significantly influences our understanding of differentiation between Golden‐winged and Blue‐winged Warblers, and of the factors that may drive speciation. To assess hybrid fitness, we used data for only males because they are usually caught near singing posts, which are used by both mated and unmated males, and which seems to provide an unbiased sample of pairing frequency. We exclude females who are most often caught in nets placed near a known nest, which would provide a biased sample of pairing frequency. Ficken and Ficken (1968a, 1968b) compiled data from several sources that showed a significant difference in the ratio of paired to unpaired males for “pures” versus hybrid: 93% (*n* = 32:3) vs. 46% (*n* = 6:7) (chi‐square = 11.781, *p* = .018). Confer and Tupper (2000) found that only 1 of 13 resident male Brewster's Warblers formed a social pair. Experimental manipulation of plumage pattern (Leichty & Grier, 2006) showed reduced pairing success for hybrid‐looking males. For our pooled results for males, hybrid fitness was significantly lower with a 35% reduction in pairing success rate for hybrids compared to Golden‐winged Warblers. Vallender et al. (2007) analyzed male and female pairing success for a study in southeastern Ontario. Based on these data, Kramer et al. (2018) suggest that “there is little evidence of costs to producing hybrid young.” However, considering just males, the data showed a pairing success rate of 42% (55 of 132) for Golden‐winged Warblers and 18% (2 of 11) for Brewster's Warblers (Vallender et al., 2007), a 57% reduction in pairing success for hybrids compared to Golden‐winged Warblers. The trend for this data for males agrees with the significant results reported by Ficken and Ficken (1968a) and the extremely low pairing success for hybrids observed by Confer and Tupper (2000), and to the significant reduction in hybrid fitness shown by our pooled results and by the paired *t* tests for our individual studies. Collectively, the published data suggest that male hybrids have a significant loss in reproductive fitness compared to both Golden‐winged and Blue‐winged Warblers. Although our estimate of behavioral isolation is much higher than our estimate of sexual selection against hybrids (Figure 2), recent work suggests that even weak postzygotic reproductive isolating barriers can potentially play a larger role in reducing gene flow than strong prezygotic barriers (Irwin, 2020). Thus, the reduced mating success of hybrids may be just as, if not more, important to speciation in this system as behavioral isolation.

Following Parkes’ model, on average half the progeny of a backcross by genetically pure Golden‐winged or Blue‐winged warblers with a Brewster's Warbler will have the Brewster's phenotype. Our compilation shows that male Golden‐winged Warblers are about three times more likely to form a backcross social pair than male Blue‐winged Warblers. Consequently, sexual selection against hybrids has a more detrimental effect on Golden‐winged than on Blue‐winged Warblers. This difference contributes to the replacement of Golden‐winged Warblers by Blue‐winged Warblers.

Despite the near‐complete levels of reproductive isolation between Golden‐winged and Blue‐winged Warblers that we document, other studies have documented high levels of introgression (Dabrowski et al., 2005; Shapiro et al., 2004; Vallender et al., 2007) and weak genome‐wide differentiation (Toews et al., 2016) in this system. Our estimates of reproductive isolation might underestimate the actual level of gene flow between Golden‐winged and Blue‐winged Warblers. Nevertheless, our primary data analysis assessed whether divergent plumage phenotypes contribute to nonrandom mating in this system, not to an assessment of the actual levels of gene flow. The percentage of the total population composed of individuals with hybrid phenotypes averaged across sites (5.2%; lower 95% CI: 4.1; upper 95% CI: 6.7) is reasonably consistent with the probability of gene flow (estimated as 1 − total RI) based on the joint effects of behavioral isolation and sexual selection against hybrids averaged across sites (3.4%; lower 95% CI: 1.1%; upper 95% CI: 5.8%).

## 5 CONCLUSIONS

The taxonomy of Golden‐winged and Blue‐winged Warblers has been debated for well over a century, since the initial description of Brewster's and Lawrence's Warblers (Brewster, 1874; Herrick, 1874). The intensity of this debate has heightened recently, in light of genomic analyses (Toews et al., 2016) whereby genetic differences between Golden‐winged and Blue‐winged Warblers are associated primarily with genes that control feather attributes. Indeed, the low level of genome‐wide divergence has been cited as evidence that Golden‐winged and Blue‐winged Warblers may be plumage morphs of a single‐species complex (Kramer et al., 2018). Alternatively, our work suggests these genomic differences relate to plumage divergence that in turn leads to high levels of reproductive isolation. We document strong behavioral isolation and significant sexual selection against hybrids, which together provide a value of 0.960 for reproductive isolation. These attributes support the view that Golden‐winged and Blue‐winged Warblers are distinct species under the biological species concept.

The taxonomic treatment of these lineages is especially important given the conservation challenges that Golden‐winged and Blue‐winged Warblers face (Sauer et al., 2017). Extensive population decline in the Golden‐winged Warbler has resulted in a petition for listing on the Endangered Species Act (Sewell, 2010), and the decision regarding listing will be highly influenced by one's interpretation of speciation in the *Vermivora* spp. complex. Ecological and genetic interactions between Blue‐winged and Golden‐winged Warblers as the former moves into sympatry with the latter appear to be a major cause of the decline of Golden‐winged Warblers (Gill, 1980; Confer et al., 2011; Rohrbaugh et al., 2016; Rosenberg et al., 2016). The final genetic outcome of this expansion is unclear. On the one hand, introgression (Vallender et al., 2009; Wood et al., 2016) may prevent further divergence between lineages (Karrenberg et al., 2019; Nosil, Harmon, & Seehausen, 2009; Sambatti, Strasburg, Ortiz‐Barrientos, Baack, & Rieseberg, 2012; Strasburg & Rieseberg, 2008), while sexual selection against hybrids may enhance isolation. Despite the high levels of reproductive isolation between Golden‐winged and Blue‐winged Warblers we document, we suggest that their continued existence may require conservation efforts that maintain or repair eco‐geographic isolation (Roth, Rohrbaugh, Will, & Buehler, 2019).

## CONFLICT OF INTEREST

There is no conflict of interest with this manuscript for any of the authors.

## AUTHOR CONTRIBUTIONS

**John L. Confer:** Conceptualization (lead); formal analysis (lead); writing – original draft (lead); writing – review and editing (lead). **Cody Porter:** Data curation (equal); formal analysis (equal); writing – original draft (equal). **Kyle R. Aldinger:** Data curation (equal); investigation (equal); writing – review and editing (supporting). **Ronald A. Canterbury:** Data curation (equal); investigation (equal); writing – review and editing (supporting). **Jeffery L. Larkin:** Data curation (supporting); investigation (supporting); supervision (supporting); writing – review and editing (supporting). **Darin J. Mcneil:** Data curation (equal); formal analysis (supporting); writing – original draft (supporting); writing – review and editing (equal).

## Data Availability

All data in this paper have been archived in Dryad. The DOI is https://doi.org/10.5061/dryad.rfj6q577f. As of 04/24/2020 the data is being curated.
